# Disease-associated regulation of gene expression by resveratrol: Special focus on the PI3K/AKT signaling pathway

**DOI:** 10.1186/s12935-022-02719-3

**Published:** 2022-09-30

**Authors:** Soudeh Ghafouri-Fard, Zahra Bahroudi, Hamed Shoorei, Bashdar Mahmud Hussen, Seyedeh Fahimeh Talebi, Sadia Ghousia Baig, Mohammad Taheri, Seyed Abdulmajid Ayatollahi

**Affiliations:** 1 Department of Medical Genetics, School of Medicine, Shahid Beheshti University of Medical Sciences, Tehran, Iran; 2 Department of Anatomical Sciences, Faculty of Medicine, Tabriz University of Medical Sciences, Tabriz, Iran; 3 Department of Anatomical Sciences, Faculty of Medicine, Birjand University of Medical Sciences, Birjand, Iran; 4 Department of Pharmacognosy, College of Pharmacy, Hawler Medical University, Erbil, Kurdistan Region Iraq; 5 Center of Research and Strategic Studies, Lebanese French University, Erbil, Kurdistan Region Iraq; 6 Department of Pharmacology, College of Pharmacy, Birjand University of Medical Sciences, Birjand, Iran; 7 Department of Pharmacology, Faculty of Pharmacy, University of Karachi, Karachi, Pakistan; 8 Institute of Human Genetics, Jena University Hospital, Jena, Germany; 9 Urology and Nephrology Research Center, Shahid Beheshti University of Medical Sciences, Tehran, Iran; 10 Phytochemistry Research Center, Shahid Beheshti University of Medical Sciences, Tehran, Iran

**Keywords:** Resveratrol, Gene expression, PI3K/AKT pathway, NF-κB, Notch

## Abstract

Resveratrol (3,5,4′-trihydroxy-*trans*-stilbene) is a natural phenol that is present in the skin of the grape, blueberry, raspberry, mulberry, and peanut. This substance is synthesized in these plants following injury or exposure to pathogens. Resveratrol is used as a dietary supplement for a long time and its effects have been assessed in animal models of human disorders. It has potential beneficial effects in diverse pathological conditions such as diabetes mellitus, obesity, hypertension, neoplastic conditions, Alzheimer's disease, and cardiovascular disorders. Notably, resveratrol has been found to affect the expression of several genes including cytokine coding genes, caspases, matrix metalloproteinases, adhesion molecules, and growth factors. Moreover, it can modulate the activity of several signaling pathways such as PI3K/AKT, Wnt, NF-κB, and Notch pathways. In the current review, we summarize the results of studies that reported modulatory effects of resveratrol on the expression of genes and the activity of signaling pathways. We explain these results in two distinct sections of non-neoplastic and neoplastic conditions.

## Introduction

**Fig. 1:**
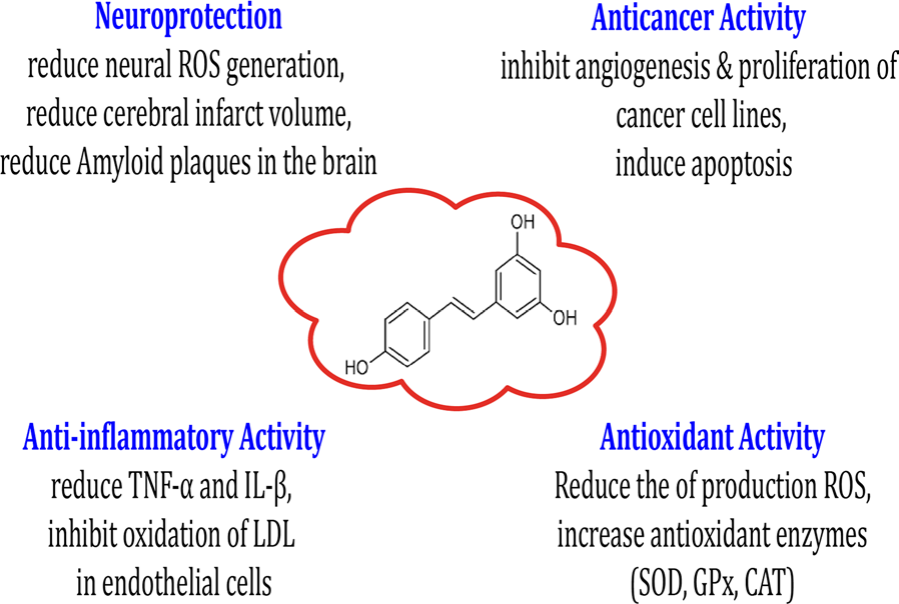
Chemical structure of resveratrol. It has been reported that resveratrol has many therapeutic effects [10–13]

Resveratrol (3,5,4′-trihydroxy-*trans*-stilbene) is a natural phenol that is synthesized by numerous plants following injury or exposure to pathogens [1]. The skin of the grape, blueberry, raspberry, mulberry, and peanut is regarded as a source of resveratrol [2]. Resveratrol is used as a dietary supplement and its effects have been assessed in animal models of human disorders (Fig. 1). Resveratrol is a pan-assay interference agent that makes positive impacts in various laboratory tests [3]. These effects are mediated through its interactions with biomolecules on cell membranes [4]. In plants, resveratrol is synthesized by the enzyme resveratrol synthase [5].

In humans, resveratrol can be administered through buccal delivery being absolved via the saliva. Yet, buccal delivery is not an efficient route since it has low aqueous solubility [6]. Moreover, high amounts of hepatic glucuronidation and sulfonation further limit the bioavailability of resveratrol [7]. Resveratrol is glucuronidated and sulfonated in the intestinal and hepatic tissues. Its sulfonation in the intestine is induced by microbial activity [8]. While the half-life of resveratrol is about 8–14 min, sulphate and glucuronide resveratrol metabolites have half-lives of more than 9 h [9].

This agent has been found to alter the expression of several genes in different pathological conditions. In the current review, we summarize the results of studies that reported modulatory effects of resveratrol on the expression of genes and the activity of signaling pathways. We explain these results in two distinct sections of non-neoplastic and neoplastic conditions. The main focus of this manuscript is on studies that reported modulatory effects of resveratrol on PI3K/AKT signaling pathway.

## Effects of resveratrol on gene expression in non-neoplastic conditions

### Cardiac diseases

In order to assess the protective effects of resveratrol against cardiac hypertrophy, Guan et al. have exposed male rats to Male rats were exposed to chronic intermittent hypoxia (CIH). CIH has resulted in the elevation of heart weight/body weight and left ventricle weight/body weight ratios as well as left ventricular remodeling. Moreover, authors have reported elevation of the apoptosis index, up-regulation of oxidative biomarkers, increase in autophagy marker Beclin-1, and down-regulation of p62 in the CIH group. Intragastric administration of resveratrol has enhanced cardiac function, amended cardiac hypertrophy, and reversed CIH-induced changes in oxidative stress and apoptosis. Mechanistically, PI3K/AKT-associated suppression of the mTOR pathway has been identified as the mediator of effects of resveratrol autophagy activation following CIH stimulation [14]. In an experiment in aged rats, Lin et al. have shown swimming exercise training, resveratrol treatment, or a combination of both can improve heart function. Authors have also reported a slight increase in the activity of the PI3K/AKT pathway in rats subjected to exercise training and resveratrol treatment. Yet, the activity of SIRT1 in the aged rat hearts has been only with resveratrol treatment. Besides, rats exposed to both interventions exhibited activation of both SIRT1 and PI3K/AKT pathways and inhibition of FOXO3 accumulation [15]. Table 1 describes the impact of resveratrol on the expression of genes in the context of cardiovascular disorders.

**Table 1:** Impact of resveratrol on the expression of genes in the context of cardiovascular disorders

Type of disease	Dose range	Cell line	Target	Pathway	Function	Refs.
*In vivo studies*						
Cardiac Hypertrophy	30 mg/kg	–	Bax, Bcl-2, Beclin-1, p62	PI3K/AKT/mTOR	RVT by targeting the PI3K/AKT/mTOR pathway could prevent chronic intermittent hypoxia-induced cardiac hypertrophy	[14]
Cardiovascular Diseases	15 mg/kg	–	SIRT1, FOXO3, Fas, FADD, Caspase-3/8, Sirt-1, BNP, TNF-α, PARP	PI3K/AKT	RVT via synergetic activation of PI3K/AKT and SIRT1signaling could improve the beneficial effects of exercise training in aging rat hearts	[15]
Heart Failure (HF)	2.5 mg/kg	–	Caspase-3, Serca2a, PLB	PI3K/AKT/eNOS	RVT via the PI3K/AKT/eNOS pathway could decrease reduces atrial fibrillation susceptibility in HF	[16]
*In vitro studies*						
Acute Myocardial Infarction (AMI)	20 μM	Cardiomyocyte	–	P13K/AKT/e-NOS	RVT via blocking the P13K/AKT/e-NOS pathway could protect cardiomyocyte apoptosis induced by I/R injury in AMI	[17]

Based on the anti-thrombotic and anti-inflammatory effects of resveratrol, this agent is also suggested to decreases COVID-19-associated mortality, which is due to activation of thrombotic and inflammatory cascades [18].

### Central nervous system (CNS) disorders

Resveratrol has been found to have neuroprotective effects against early brain injury (EBI) following subarachnoid hemorrhage (SAH). Experiments in rat models have shown that intraperitoneal administration of this agent decreases mortality and brain edema following SAH. Moreover, resveratrol has enhanced neurological scores in these animals. Histological studies have shown the effect of resveratrol in the reduction of neuronal pyknosis and swelling. Moreover, resveratrol has enhanced expressions of beclin-1, LC3-II, LC3-II/LC3-I, and Bcl-2, while decreasing p-AKT, p-mTOR, p62, cleaved caspase-3, caspase-9, and BAX levels. Further studies have verified the effects of resveratrol in the induction of autophagy. Therefore, the neuroprotective effect of resveratrol is exerted through the regulation of autophagy and apoptosis via modulating the AKT/mTOR pathway [19].

**Fig. 2:**
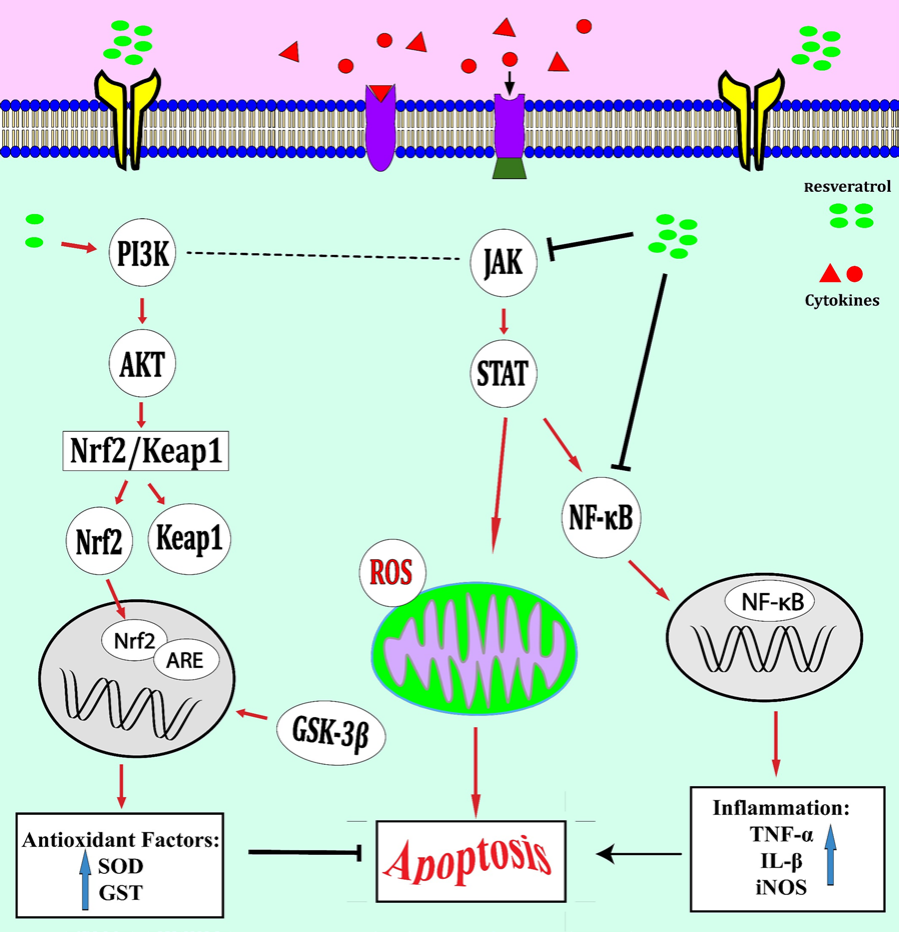
Resveratrol could activate the PI3K/AKT pathway [25]. On the other hand, this mentioned pathway could increase the Nrf2 translocation, finally induce transcription of anti-oxidative enzymes involved in inhibiting apoptosis. Moreover, GSK-3β could inhibit the Nrf2-ARE, then the transcription of antioxidant enzymes is induced. Interestingly, resveratrol by inactivating JAK-STAT or the NF-kB pathways could decrease ROS production and cell death [34, 35]

Neuroprotective effects of resveratrol have also been investigated in a rat model of middle cerebral artery occlusion. Resveratrol has remarkably enhanced neurological function, decreased cerebral infarct size, reduced neuron injury, and diminished neuron apoptosis. Mechanistically, resveratrol up-regulates p-JAK2, p-STAT3, p-AKT, p-mTOR, and BCL-2 levels, while down-regulating cleaved caspase-3 and BAX levels. Taken together, resveratrol protects against cerebral ischemia/reperfusion injury through induction of the activities of JAK2/STAT3 and PI3K/AKT/mTOR pathways [20]. Another experiment has shown that resveratrol reduces neurological deficit scores and MPO activity and suppresses induction of IL-1β, TNFα, and COX2 inflammatory markers. In addition, resveratrol attenuates ischemic brain injury following cerebral artery occlusion via modulation of PI3K/AKT signaling pathway [21] (Fig. 2). Through upregulating heme oxygenase-1 (HO-1) via the PI3K/AKT/Nrf2 axis, resveratrol can attenuate the cytotoxic effects of amyloid-β1–42 in PC12 cells [22]. Moreover, through activating PP2A and PI3K/AKT induced-inhibition of GSK-3β, resveratrol can inhibit Tau phosphorylation in the rat brain [23]. Thus, resveratrol may be considered as an anti-Alzheimer's disease substance. Table 2 describes the impact of resveratrol on the expression of genes in the context of CNS disorders.

**Table 2:** Impact of resveratrol on the expression of genes in the context of CNS disorders

Type of disease	Dose range	Cell line	Target	Pathway	Function	Refs.
*In vivo studies*						
Subarachnoid Hemorrhage (SAH)	60 mg/kg	–	Beclin-1, LC3-II, Bcl-2, p62, Caspase-3/9	AKT /mTOR	RVT via downregulating AKT/mTOR pathway could promote the autophagy process in SAH model rats	[19]
Cerebral Ischemia Injury (CII)	30 mg/kg	–	BcL-2, Bax, Caspase-3	JAK2/STAT3, PI3K/AKT/mTOR	RVT via activating JAK2/STAT3/PI3K/AKT/mTOR pathway could provide neuroprotection against cerebral I/R injury	[20]
CII	100 mg/kg	–	IL-1β, TNFα, COX2	PI3K/AKT	RVT via activating PI3K/AKT pathway could attenuate brain damage in permanent focal cerebral ischemia	[21]
CII	30 mg/kg	–	–	AKT/GSK-3β	RVT via regulating the AKT/GSK-3β pathway could improve neuronal damage against MCAO-induced CII	[24]
CII	20 mg/kg	–	GSK-3β, DJ-1, PTEN, Nrf-2, Bax, Caspase-3, Bcl-2	PI3K/AKT,	RVT via reducing of DJ-1 expression and activating of PI3K/AKT/GSK-3β pathway could contribute to post I/R cerebral damage	[25]
Chronic cerebral hypoperfusion (CCH)	50 mg/kg	–	Caspase-3, Bcl-2, Bax, LC3B, 4E-BP1, Beclin-1, S6K1	PI3K/AKT/mTOR	RVT via the AKT/mTOR pathway could Improve cognitive dysfunction in rats with CCH	[26]
Alzheimer’s Disease (AD)	0–40 μM	PC12	HO1	PI3K/AKT/Nrf2	RVT by upregulating heme oxygenase-1 (HO-1) via the PI3K/AKT/Nrf2 axis could attenuate the cytotoxicity induced by amyloid-β1–42 in PC12 cells	[22]
AD	300 mg/kg	–	PP2A, GSK-3β, Tau, Caspase-3, Bcl2, Bax	PI3K/AKT, AMPK	RVT via activating PP2A and PI3K/AKT induced-inhibition of GSK-3β could inhibit Tau phosphorylation in rat brain	[23]
Parkinson's Disease (PD)	15–30 mg/kg	–	Bax, Bcl-2, Caspase-3, PDK1	PI3K/AKT	RVT via activating the PI3K/AKT pathway could protect dopaminergic neurons from 6-hydroxy dopamine (6-OHDA)-induced apoptosis	[27]
Spinal Cord Injury (SCI)	100 mg/kg, 40 μM	Primary microglia, neurons	Beclin-1, Caspase-3, LC3B	PI3K	RVT-primed exosomes via the PI3K pathway could promote the recovery of motor function in SCI rats	[28]
*In vitro studies*						
Intervertebral Disc Degeneration (IVDD)	200 mM	NPCs	Caspase-3, NF-κB, GSK-3β	PI3K/AKT/mTOR	RVT and 17β-estradiol via The PI3K/AKT/GSK-3β and PI3K/AKT/mTOR pathways could prevent IL-1β induced apoptosis in the human nucleus pulposus	[29]
IVDD	10–200 μM	NPCs	Caspase-3, MMP-3, MMP-13, COL2a-1, Aggrecan	PI3K/AKT	RVT and 17β-estradiol via the PI3K/AKT/caspase-3 pathway could play a role in apoptosis induced by interleukin-1β in rat nucleus pulposus cells	[30]
IVDD	50–100 μM	NP	GAPDH, SOX9, Aggrecan, Collagen II	PI3K/AKT	RVT via activating the PI3K/AKT pathway could increase nucleus pulposus matrix synthesis	[31]
IVDD	50 μM	NP	Aggrecan, Collagen II, Beclin-1, LC3	PI3K/AKT	RVT via the PI3K/AKT pathway by activating autophagy could enhance matrix biosynthesis of nucleus pulposus cells	[32]

A clinical trial in patients with Alzheimer's disease has shown measurable levels of resveratrol and its major metabolites in plasma and cerebrospinal fluid of patients following treatment with this substance. However, brain volume loss has been promoted by treatment with resveratrol [33].

### Diabetic complications

The beneficial effects of resveratrol on cardiac function have been assessed in an animal model of diabetic cardiomyopathy. Resveratrol has suppressed high glucose-associated apoptosis of ventricular myocytes in neonatal rats. Moreover, resveratrol has reversed the effects of high glucose in reduction of cell viability, inhibition of AKT and FoxO3a phosphorylation, and suppression of cytoplasmic transfer of FoxO3a. The protective effects of resveratrol have been abolished by a PI3K inhibitor, indicating that the therapeutic effect of this agent is mediated through inhibition of apoptosis via the PI3K/AKT/FoxO3a cascade [36]. Another study has shown that resveratrol through up-regulating mmu-miR-363-3p via the PI3K/AKT pathway can reverse high-fat diet-induced insulin resistance [37]. Resveratrol has also shown protective effects against high glucose-associated apoptosis and senescence of nucleus pulposus cells. Functionally, resveratrol inhibits the production of reactive oxygen species (ROS) and activates PI3K/AKT pathway under the high glucose condition [38]. The protective effects of resveratrol against diabetic nephropathy are exerted through modulation of PI3K/AKT/FoxO3a pathway, attenuation of the high glucose-induced oxidative stress, and reduction of apoptosis [39]. Resveratrol-induced suppression of PKC expression has also been shown to counteract NOX-associated endothelial to mesenchymal transition in endothelial cells of retina following exposure to high glucose [40]. Table 3 describes the impact of resveratrol on the expression of genes in the context of diabetic complications.

**Table 3:** Impact of resveratrol on the expression of genes in the context of diabetic complications

Type of disease	Dose range	Cell line	Target	Pathway	Function	Refs.
*In vivo studies*						
Diabetic cardiomyopathy (DCM)	5–50 mg/kg, 10 μM	Ventricular myocytes	Bax, Bcl-2, Histone H3	PI3K/AKT/FoxO3a	RVT via the PI3K/AKT/FoxO3a pathway by inhibiting apoptosis could ameliorate cardiac dysfunction in DCM	[36]
Type 1 diabetes (T1D)	40 mg/kg	–	GSK-3β, PTEN, Nrf2, NQO-1, HO-1, p62, Caspase-3, LC3II, Keap1	AKT	RVT by AKT-mediated Nrf2 activation via p62-dependent Keap1 degradation could reduce testicular apoptosis in T1D mice	[41]
Type 2 diabetes	100 mg/kg, 0–100 μM	HepG2	miR-363-3p, FOXO1, G6PC	PI3K/AKT	RVT by upregulating mmu-miR-363-3p via the PI3K/AKT pathway could reverse high-fat diet (HFD)-induced insulin resistance	[37]
Neuropathic pain	40 mg/mL	–	SIRT1/PGC1α	PI3K/AKT	RVT via PI3K/AKT and SIRT1/PGC1α pathways could inhibit paclitaxel-induced neuropathic pain	[42]
Diabetic nephropathy (DN)	10 mg/kg, 25 μM	Rat Mesangial Cell (RMC)	PAI-1	AKT/NF-κB p65	RVT via inhibiting AKT/NF-κB pathway could prevent mesangial cell proliferation and diabetes-induced renal inflammation	[35]
*In vitro studies*						
DN	10 μM	PC12	Bim, FoxO3a	PI3K/AKT	RVT via the PI3K/AKT/FoxO3a pathway could attenuate the HG-induced oxidative stress and apoptosis in PC12 cells	[39]
Diabetes mellitus	100 μM	NP	Caspase-3, Bcl-2, Bax, p53	PI3K/AKT	RVT via activating PI3K/AKT pathway could attenuate high glucose-induced NP cell senescence and apoptosis	[38]

### Gastrointestinal disorders

Resveratrol has been shown to exert protective effects against radiation-induced intestinal damage. This agent has amended the intestinal oxidative stress markers, malondialdehyde and glutathione levels, and enzymatic activity of catalase. Additionally, resveratrol has decreased the production of proinflammatory molecules TNF-α, NF-κB, and IL-1β in the intestine. These effects have been accompanied by down-regulation of PI3K, AKT, and mTOR in the intestinal tissue of irradiated animals. Therefore, resveratrol can be used as a potential adjuvant in radiotherapeutic regimens [43]. Moreover, resveratrol via the PI3K/AKT-mediated Nrf2 pathway could protect intestinal cells against oxidative stress [44]. The protective effects of resveratrol against liver fibrosis have been verified in different studies. Resveratrol can regulate the activity of hepatic stellate cells via modulating NF-κB and PI3K/AKT pathways [45]. Moreover, resveratrol via the miR-20a-mediated activation of the PTEN/PI3K/AKT pathway can inhibit LF [46]. Table 4 describes the impact of resveratrol on the expression of genes in the context of gastrointestinal disorders.

**Table 4:** Impact of resveratrol on the expression of genes in the context of gastrointestinal disorders

Type of disease	Dose range	Cell line	Target	Pathway	Function	Refs.
*In vivo studies*						
Intestinal Injury	20 mg/kg	–	TNF-α, NF-κB, IL-1β	PI3K/AKT/mTOR	RVT via modulating PI3K/AKT/mTOR pathway could reduce intestinal inflammation in irradiated rats	[43]
Liver Fibrosis (LF)	40–200 mg/kg, 10–50 mg/mL	HSC-T6	miR-20a, α-SMA, TIMP-1, TGF-β1, LC3-II, LC3-I, Beclin1, Atg7	PTEN/PI3K/AKT	RVT via the miR-20a-mediated activation of the PTEN/PI3K/AKT pathway can inhibit LF	[46]
LF	20–50 mg/kg, 0–125 μg/mL	LX-2	α-SMA, Collagen-I, IκB-α, P65	AKT, NF-κB	RVT via the AKT/NF-κB pathways could attenuate the progression of LF	[47]
*In vitro studies*						
Intestinal Damage	0–50 μM	IPEC-J2, 293 T	Claudin-1, Occludin, ZO-1, Keap1, NFE2L2, SOD-1, HO-1, CAT, GSX-1, Nrf2	PI3K/AKT	RVT via the PI3K/AKT-mediated Nrf2 pathway could protect IPEC-J2 cells against oxidative stress	[44]
Hepatic Fibrosis	3.125, 6.25, 12.5 μM	T-HSC/Cl-6	Collagen-I, α-SMA, TLR4, M8, LXR-α, LXR-β	PI3K/AKT, NF-κB	RVT via modulating NF-κB and the PI3K/AKT pathway could regulate activated hepatic stellate cells (HSCs)	[45]

### Other disorders

Resveratrol has also been shown to inhibit ox-LDL-stimulated expression of TLR4 in activated platelets. This effect has been similarly seen in LPS-activated and puromycin-pretreated platelets. Mechanistically, resveratrol attenuates ox-LDL-stimulated phosphorylation of NF-κB and STAT3. Moreover, the suppressive impact of resveratrol on TLR4 expression has been correlated with the inhibition of phosphorylation of AKT. Combined administration of resveratrol and a PI3K inhibitor synergistically inhibits AKT phosphorylation and TLR4 expression. Besides, resveratrol has increased the expression of sirtuin 1 and phosphorylation of AMPK, which was decreased by ox-LDL. Besides, resveratrol has been shown to reduce platelet aggregation and adhesion and CD40L expression in ox-LDL-exposed platelets. Therefore, resveratrol can inhibit the TLR4-associated inflammatory responses in ox-LDL-induced platelets and might be used as an option for the treatment of thrombosis and atherosclerotic conditions [48]. In addition, a certain formulation of resveratrol-loaded nanoparticles has been shown to inhibit LPS-induced accumulation of leukocytes in the bronchoalveolar fluid. This effect has been accompanied by improvement of respiratory function, prevention of accumulation of leukocytes and neutrophils, and reduction of IL-6, KC, MIP-1α, MIP-2, MCP-1, and RANTES levels in lung tissues. Additionally, the mentioned formulation could inhibit MDA levels and SOD activity and block ERK and PI3K/AKT pathways after LPS stimulation [49]. In addition, resveratrol through suppression of PI3K/Nrf2/HO-1 pathway could inhibit oxidative stress, inflammation, and cell apoptosis and alleviate acute lung injury in septic rats [50]. The protective effect of resveratrol against sepsis-induced changes in the myocardium has been shown to be exerted through suppression of NF-kB and induction of the PI3K/AKT/mTOR pathway [51]. Table 5 describes the impact of resveratrol on the expression of genes in the context of other disorders.

**Table 5:** Impact of resveratrol on the expression of genes in the context of other disorders

Type of disease	Dose range	Cell line	Target	Pathway	Function	Refs.
*In vivo studies*						
Acute Lung Injury (ALI)	2.5–10 mg/kg	–	IL-6, KC, MIP-1α, MIP-2, MCP-1, RANTES	PI3K/AKT, ERK	Delivering RVT by polymeric nanocapsules via the ERK/PI3K/AKT pathways could ameliorate LPS-induced ALI	[49]
Sepsis	30 mg/kg	–	MIP-2, IL-18, IL-10, Caspase-3	PI3K/Nrf2/HO-1	RVT via inhibiting PI3K/Nrf2/HO-1 pathway could inhibit oxidative stress, inflammation, and cell apoptosis to alleviate ALI in septic rats	[50]
Sepsis	60 mg/kg	–	IL-6, IL-1b, TLR4, Capase-3, Bax, Bcl2, NF-kB	PI3K/AKT/mTOR	RVT via inhibiting the NF-kB and activating the PI3K/AKT/mTOR pathway could protect the myocardium in sepsis	[51]
Allergic Diseases	10 mg/kg, 10–100 μM	BMMCs, FSMCs, PBMCs	IL-6, IL-13, TNF-α, NF-κB, IKKα/β, p65, P-38, Syk, Gab2	MK2/PI3K/AKT	RVT via the MK2/3–PI3K/AKT axis could inhibit IL-33–mediated mast cell activation	[52]
Osteoarthritis (OA)	45 mg/kg 50 μM	SW1353	TLR4, MyD88, TRIF, IL-1β, NF-κB p65	PI3K/AKT	RVT by inhibiting TLR4 via the activation of the PI3K/AKT pathway could inhibit the development of obesity-related OA	[53]
Chronic Unpredictable Mild Stress (CUMS)	40–80 mg/kg	–	TNF-α, IL-6, IL-1β, Bax, Bcl-2	AKT/GSK-3β	RVT via activating the AKT/GSK-3β pathway could exert a protective effect in CUMS–induced depressive-like behavior	[54]
–	100 mg/kg, 20 μM	293 T	klf5, c-Myc, Cav-1	PI3K/PKD1/AKT	RVT via inhibiting the PI3K/PKD1/AKT pathway could activate klf5 phosphorylation and then attenuate the interaction of klf5 with c-Myc	[55]
–	100 mg/kg 40–100 μM	hPASMC	Arginase I, Arginase II, Caspase-3	PI3K/AKT	RVT via the PI3K/AKT pathway could prevent hypoxia-induced arginase II expression and proliferation of hPASMC	[56]
*In vitro studies*						
Thrombosis and atherosclerosis	1–100 μM	Platelet	PECAM-1, TLR4, STAT3, NF-кB p65, Sirt1	AKT, AMPK	RVT via STAT3 and AKT pathways could suppress TLR4 activation in oxidized low-density lipoprotein-activated platelets	[48]
–	15 μmol/L	BMSCs, P3	MyoD1, Myogenin	SIRT1/AKT/FOXO1	RVT via activating the SIRT1/AKT/FOXO1 pathway could reverse myogenic induction suppression caused by high glucose	[57]
–	20 μM	Chondrocytes	Collagen-II, COX-2, PGE2, JNK, P38	AKT, ERK, MAPK	RVT via the ERK/p38/AKT pathway could regulate the differentiation and inflammation of chondrocytes	[58]

## Effects of resveratrol on gene expression in neoplastic conditions

### Hematological malignancies

Resveratrol can combat multidrug resistance (MDR) in leukemia. This substance has been shown to enhance the anti-proliferative effect of bestatin in the K562/ADR leukemia cell line. Concurrent treatment of leukemic cells with bestatin and resveratrol has decreased IC50 values of bestatin and increased activity of caspase-3 and caspase-8, indicating the potential effect of resveratrol in the enhancement of bestatin-induced apoptosis. Resveratrol has enhanced intracellular levels of bestatin via suppressing P-gp function and decreasing the expression level of P-gp, therefore increasing the anti-proliferative effect of bestatin in K562/ADR cells. Mechanistically, resveratrol has been shown to decrease AKT and mTOR phosphorylation without affecting the phosphorylation of JNK or ERK1/2 [59]. Moreover, resveratrol can regulate apoptosis and proliferation of leukemia cells through modulation of PTEN/PI3K/AKT [60]. Table 6 describes the impact of resveratrol on the expression of genes in the context of hematological malignancies.

**Table 6:** Impact of resveratrol on the expression of genes in the context of hematological malignancies

Type of cancer	Samples	Dose range	Cell line	Target	Pathway	Function	Refs.
Leukemia	In vitro	10 mM	K562/ADR, K562	P-gp, Caspase-3/8, ERK1/2, JNK	PI3K/AKT/mTOR	RVT via suppressing the PI3K/AKT/mTOR pathway could increase the anti-proliferative activity of bestatin	[59]
Leukemia	In vitro	0–20 μM	PBMCs, HL-60, NB-4	–	PTEN/PI3K/AKT	RVT via regulating the PTEN/PI3K/AKT pathway could affect apoptosis and proliferation of leukemia cells	[60]
Acute Myeloid Leukemia (AML)	In vitro	25–200 lmol/L	HL-60, HL-60/ADR	MRP1	PI3K/AKT/Nrf2	RVT via the PI3K/AKT/Nrf2 Pathway could reverse the drug resistance of AML HL-60/ADR cells	[61]
Chronic Myeloid Leukemia (CML)	In vitro	60 μM	K562	p70S6K, 4EBP1, Cyclin-D1, Caspase-3,	PI3K/AKT/mTOR	RVT via downregulating the PI3K/AKT/mTOR pathway could play a role in the apoptosis of K562 cells	[62]

### Gastrointestinal cancers

Resveratrol has protective effects against bile acid-induced gastric intestinal metaplasia. Resveratrol has been shown to decrease the expression of CDX2 and enhance the activity of FoxO4 in gastric cell lines. Based on the bioinformatics and chromatin-immunoprecipitation analyses, FoxO4 has been shown to bind with the promoter region of CDX2. These effects are mediated through the enhancement of nuclear translocation phospho-FoxO4. In addition, resveratrol enhances FoxO4 phosphorylation via modulation of the PI3K/AKT pathway. Taken together, resveratrol can decrease bile acid-induced gastric intestinal metaplasia via the PI3K/AKT/p-FoxO4 cascade. Thus, it has a protective effect against bile acid-induced gastric intestinal metaplasia particularly those associated with bile acid reflux [63]. In addition, through regulating the PTEN/ PI3K/AKT pathway, resveratrol could induce cell cycle arrest in human gastric cancer cells [64]. Besides, via MARCH-1-induced regulation of the PTEN/AKT pathway, resveratrol can inhibit the malignant progression of hepatocellular carcinoma [65]. Resveratrol can also up-regulate connexin43 and inhibit the AKT pathway, therefore sensitizing colorectal cancer cells to cetuximab [66]. Table 7 describes the impact of resveratrol on the expression of genes in the context of gastrointestinal cancers.

**Table 7:** Impact of resveratrol on the expression of genes in the context of gastrointestinal cancers

Type of cancer	Dose range	Cell line	Target	Pathway	Function	Refs.
*In vivo studies*						
Gastric cancer (GC)	50 mg/kg, 10–200 mg/L	SGC7901, SGC7901/DOX, MGC803	TSC1, TSC2, p70S6K, Caspase-3/9, Vimentin, E-cadherin	PTEN/AKT, mTOR	RVT via modulating PTEN/AKT pathway by inhibiting EMT could reverse doxorubicin resistance in GC	[67]
Hepatocellular Carcinoma (HCC)	0–100 mg/kg, 20–80 μM	HepG2, Hep3B	MARCH-1, STAT3, VEGF, Bcl-2	PTEN/AKT	RVT via MARCH-1-induced regulation of the PTEN/AKT pathway and inhibit malignant progression of HCC	[65]
Colorectal Cancer (CRC)	1 mg/kg 5 μg/mL	HCT116, CT26	Cx43, EGFR, NF-kB p65, IKKa, IkBa,	AKT, PI3K, mTOR, MAPK	RVT via upregulating connexin43 and inhibition of the AKT pathway could sensitize CRC cells to cetuximab	[66]
CRC	50–150 mg/kg , 0–80 μM	HCT116, SW480	PCNA, Caspase-3, GSK-3β,	PTEN/PI3K/AKT, Wnt/β-catenin	RVT via the Wnt/β-catenin and PTEN/PI3K/AKT pathways could play a role in human colon cancer cell proliferation	[68]
CRC	150 mg/kg, 0–240 μmol/L	SW480 and SW620	N-cadherin, E-cadherin, Vimentin	AKT/GSK-3β/Snail	RVT via the AKT/GSK‑3β/Snail pathway could inhibit the metastasis and invasion of CRC cells	[69]
*In vitro studies*						
Gastric intestinal metaplasia (GIM)	200 μM	GES-1, AGS, BGC823, SGC7901, MKN45, MKN28, AZ521, HCT116	CDX2, Villin1, Klf4, Cadherin17, Muc2	PI3K/AKT/p-FoxO4	RVT via the PI3K/AKT/p-FoxO4 pathway could inhibit bile acid-induced GIM	[63]
GC	50–200 μmol/L	MGC803	GSK3β, Cyclin-D1	PTEN/ PI3K/ AKT	RVT via regulating the PTEN/ PI3K/AKT pathway could induce cell cycle arrest in human gastric cancer MGC803 cells	[64]
HCC	0–200 μM	HepG2	FoxO3a/Bim	AKT	RVT via modulating AKT/FoxO3a/Bim pathway could induce apoptosis in HepG2 cells	[70]
HCC	100 μM	HepG2, Bel-7402, SMMC-7721	SIRT1, Bcl-2, Caspase-3/7, PARP, PCNA, Bax	PI3K/AKT	RVT via SIRT1 mediated post-translational modification of PI3K/AKT signaling could inhibit migration and proliferation in HCC cells	[71]
CRC	10–40 μM	DLD1, HCT15	Cyclin-D1, Cyclin-E2, Bcl-2, p53, Bax	AKT/STAT3	RVT via targeting the AKT/STAT3 pathway could suppress colon cancer growth	[72]
CRC	40–60 μM	HCT116, 293 T	BMP7, GFP, PTEN, BAD, Bcl-2, Smad1/5/8	PI3K/AKT	RVT via upregulating BMP7 could inactivate PI3K/AKT signaling in human colon cancer cells	[73]

Concentration of resveratrol and its metabolites has been assessed in the colorectal tissues of humans who received resveratrol in a clinical study on colorectal cancer patients who took eight daily doses of resveratrol at 0.5 or 1.0 g prior to surgical resection of tumors. This study ahs confirmed tolerability of resveratrol. More importantly, these doses of resveratrol have been shown to produce sufficient concentrations for induction of anti-cancer effect in the gastrointestinal tract [74]

### Reproductive system cancers

Resveratrol has been shown to decrease expression levels of MTA1, a constituent of the nucleosome remodeling and deacetylating (NuRD) complex which is up-regulated in numerous malignancies [75]. Moreover, resveratrol can enhance acetylation and reactivation of PTEN through suppression of the MTA1/HDAC complex, leading to blockage of the AKT pathway. Further experiments in the orthotopic model of prostate cancer have verified the effects of resveratrol in the enhancement of PTEN expression, reduction of p-AKT levels, in suppression of proliferation. Therefore, resveratrol can decrease the activity of survival pathways of prostate cancer via modulating the MTA1/HDAC axis [76]. In ovarian cancer cells, resveratrol can induce apoptosis and impair glucose uptake via AKT/GLUT1 axis [77]. Moreover, resveratrol has been shown to induce cell death via ROS‑dependent inactivation of Notch1/PTEN/AKT cascade [78]. Table 8 describes the impact of resveratrol on the expression of genes in the context of reproductive system cancers.

**Table 8:** Impact of resveratrol on the expression of genes in the context of cancers of the reproductive system

Type of cancer	Dose range	Cell line	Target	Pathway	Function	Refs.
*In vivo studies*						
Prostate Cancer (PCa)	50 mg/kg 5–100 μM,	DU145, PC3M, 293 T	MTA1, HDAC, ERK1/2, HDAC1, HDAC2, Lamin-A, myc, Flag	PTEN, AKT	RVT by regulating the PTEN/AKT pathway via inhibiting the MTA1/HDA unit could affect the progression and survival pathways of prostate cancer	[76]
*In vitro studies*						
PCa	25–200 μM	LNCaP, RWPE-1, LNCaP-B	ARV7, Bax, Bcl-2, AR	PI3K/AKT	RVT via PI3K/AKT pathway and ARV7 could promote apoptosis in LNCaP prostate cancer cells	[79]
PCa	0–50 μM	PC-3	E-cadherin, Vimentin, Bax, Bcl-2, Caspase-3/9	PI3K/AKT	RVT via downregulating the PI3K/AKT pathway could suppress the EMT in PC-3 cells	[80]
Ovarian Cancer	50 mM	PA-1, OVCAR3, MDAH2774, SKOV3, PBMC, RBC, OSE1, OSE2	P70s6K, mTOR, 4EBP1, GLUT2, GLUT3, GLUT4, GLUT1	AKT	RVT via AKT/GLUT1 axis could induce apoptosis in ovarian cancer cells by impairing glucose uptake	[77]
Ovarian Cancer	0‑200 μM	A2780, SKOV3	Caspase-3	Notch1/PTEN/AKT	RVT via notch1/PTEN/AKT signaling could induce cell death in ovarian cancer cells	[78]

A phase I clinical study in the prostate cancer pathogenesis has demonstrated potential use of resveratrol could for delaying cancer recurrence. Pulverized muscadine grape skin which comprises resveratrol could delay recurrence of prostate cancer through increasing the PSA doubling time. Yet, the obtained results have not been statistically significant [81].

### Lung cancer

Resveratrol has been shown to inhibit the expression of XRCC1 and increase the etoposide-associated apoptosis in non-small cell lung cancer (NSCLC) cells. Thus, the inhibitory role of resveratrol on the expression of XRCC1 improves the sensitivity of these cells to etoposide [82]. Moreover, through suppressing the PI3K/AKT-HK2 pathway, resveratrol can play a role in the clinical prevention and treatment of NSCLC [47]. Resveratrol also activates SIRT1 and stimulates protective autophagy in NSCLC cells through suppression of AKT/mTOR and induction of p38-MAPK [83]. Finally, resveratrol can sensitize lung cancer cells to TRAIL via suppressing the AKT/NF-κB pathway [84]. Table 9 describes the impact of resveratrol on the expression of genes in the context of lung cancer.

**Table 9:** Impact of resveratrol on the expression of genes in the context of lung cancer

Type of cancer	Dose range	Cell line	Target	Pathway	Function	Refs.
*In vivo studies*						
Non-Small Cell Lung Cancer (NSCLC)	30 mg/kg 0–100 μM	H460, H1650, HCC827	HK2, Caspase-3, PARP,	AKT, ERK1/2, EGFR	RVT via suppressing the PI3K/AKT-HK2 pathway could play a role in the clinical prevention and treatment of NSCLC	[47]
*In vitro studies*						
NSCLC	25–200 μM	H1703, H1975	XRCC1	AKT, ERK1/2	RVT via downregulating ERK1/2 and AKT-mediated XRCC1 could enhance the chemosensitivity to etoposide in NSCLC cells	[82]
NSCLC	200 μM	A549, H1299	Beclin-1, LC3 II/I, SIRT1, P62, p70S6K	AKT/mTOR, p38-MAPK	RVT by activating p38-MAPK and inhibiting the AKT/mTOR pathway could induce protective autophagy in NSCLC	[83]
NSCLC	0–50 μM	A549, HCC-15	LC3-II, P62, p53, Bax, Bcl-2, Bcl-xl, Caspase-3/8, PUMA, Cytochrome-c	AKT, NF-κB	RVT via suppressing the AKT/NF-κB pathway could sensitize lung cancer cells to TRAIL	[84]
Small Cell Lung Cancer (SCLC)	40 μg/mL	H446	c-Myc, AIF, Bcl-2, Bax, Bcl-xL, Cytochrome-c	PI3K/AKT	RVT via the PI3K/AKT/c-Myc pathway could inhibit viability in SCLC H446 cells	[85]

### Other cancers

**Fig. 3:**
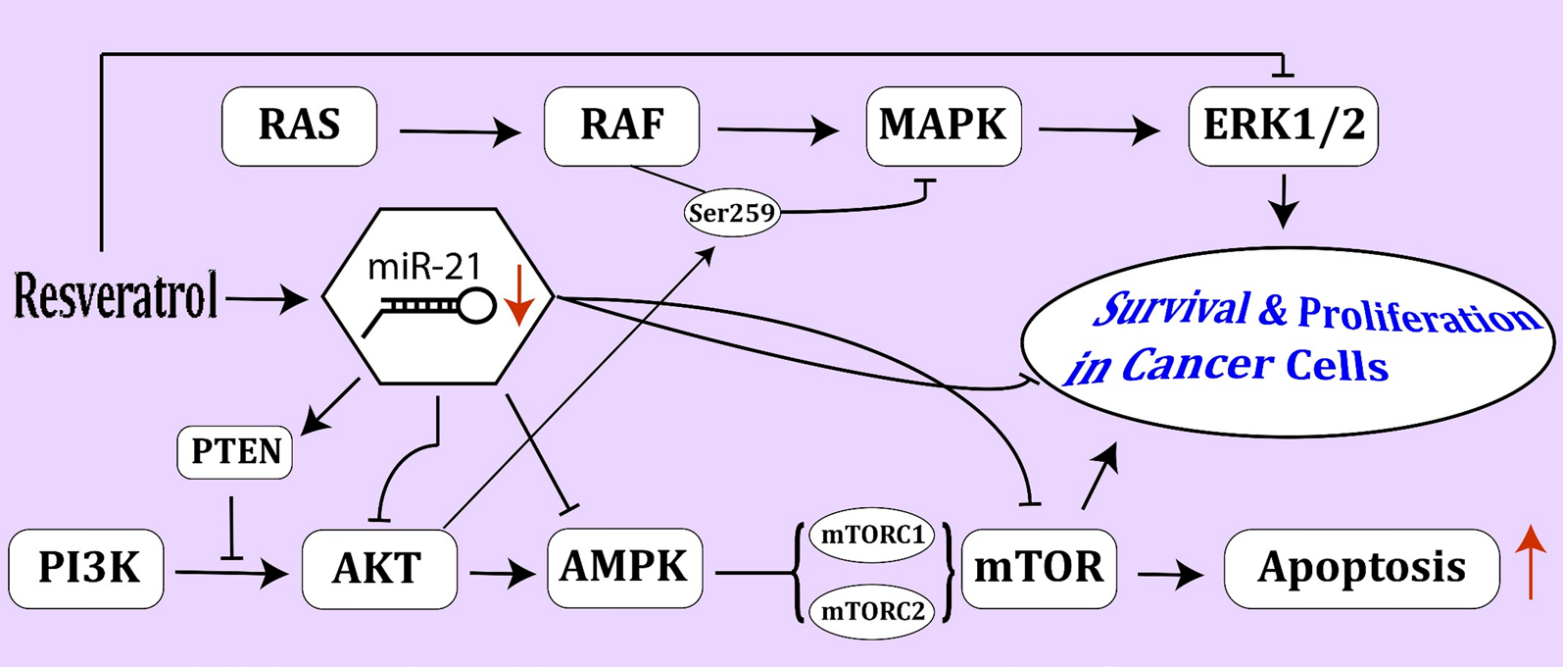
Treatment with resveratrol could decrease expression of miR-21 and finally decrease cancer cell survival; these events have been occurred after enhancing PTEN expression and blocking PI3K/AKT and mTOR pathways [94]. Also, resveratrol could decrease cancer cell survival and proliferation via inhibiting the ERK1/2 pathway [96, 100]

Resveratrol has been shown to suppress the proliferation of both parental and vemurafenib-resistant melanoma cell lines. Moreover, it can reduce AKT phosphorylation in these cells. Therefore, it can reverse vemurafenib resistance in patients receiving BRAF inhibitors [86]. Moreover, by inhibiting the PI3K/AKT/mTOR pathway, it could promote autophagy and suppress the growth of melanoma cells [87]. Resveratrol has also been shown to sensitize breast cancer cells to docetaxel-induced cytotoxicity via inhibiting docetaxel-mediated activation of the HER-2/AKT axis [88]. In addition, resveratrol can promote the anti-tumor effects of rapamycin in papillary thyroid cancer via modulation of the PI3K/AKT/mTOR pathway [89]. Table 10 describes the impact of resveratrol on the expression of genes in the context of cancers (Fig. 3).

**Table 10:** Impact of resveratrol on the expression of genes in the context of other cancers

Type of cancer	Dose range	Cell line	Target	Pathway	Function	Refs.
*In vivo studies*						
Breast cancer (BCa)	50 mg/kg, 10–200 mg/L	MCF-7/DOX, MCF-7, MDA-MB-231	Caspase-3, P70S6K	PI3K/AKT/mTOR	RVT via inhibiting PI3K/AKT/ mTOR pathway could play a role in DOX resistance in breast neoplasm	[90]
Papillary Thyroid cancer (PTC)	30 mg/kg 50 μM	KTC-1,TPC-1	Caspase-3/8/9, Bax, Bcl-xl, Mcl-1, p70S6K	PI3K/AKT/mTOR	RVT via the PI3K/AKT/mTOR pathway could promote the anti-tumor effects of rapamycin in papillary thyroid cancer	[89]
Glioblastoma multiforme (GBM)	10 mg/kg, 0–20 μM	GICs	IKKα/β, JNK, mTOR, ERK1/2, IκBα p38, MMP-2, Lamin-A, Nestin, GFAP	PI3K/ AKT/NF-κB	RVT via downregulating PI3K/AKT/NF-κB pathway could inhibit invasion of glioblastoma-initiating cells (GICs)	[91]
*In vitro studies*						
Melanoma	4 μM-18 μM	Human melanoma cell	–	AKT	RVT via dephosphorylation of AKT could overcome resistance to vemurafenib in BRAF-mutated melanoma cells	[86]
Melanoma	100 μM	B16	LC3-l, LC3-ll, Beclin-1, S6K, 4E-BP1	Ceramide/AKT/mTOR	RVT via the ceramide/AKT/mTOR pathway could trigger protective autophagy in melanoma B16 cells	[87]
Melanoma	0–100 μM	B16-F10, A375	Beclin-1, Caspase-9, P62, LC3II/I	PI3K/ AKT/mTOR	RVT via inhibiting the PI3K/AKT/mTOR pathway could promote autophagy and suppress melanoma growth	[92]
Pheochromocytoma	10–1000 μM	PC12	Caspase-3, iNOS	PI3K, AKT/p38 MAPK	RVT via AKT/p38 MAPK signaling could attenuate apoptosis, and protect neuronal cells from isoflurane-induced inflammation	[93]
BCa	10–25 μM	SK-BR-3, MCF7, T47D, MDA-MB-231	Caspase-7/8, JNK, P38, XIAP, Survivin, Bcl-2	AKT, HER-2, MAPK	RVT via inhibiting docetaxel-mediated activation of the HER-2/AKT axis could sensitize BCa cells to docetaxel-induced cytotoxicity	[88]
Bladder cancer	0–50 μmol/L	T24, 5637, SV-HUC-1	miR-21, Bcl-2, Caspase-3	AKT	RVT via miR-21 regulation of the AKT/Bcl-2 pathway could induce apoptosis of bladder cancer cells	[94]
Chondrosarcoma	25–100 μM	JJ012, SW1353	MMP2, MMP9	PI3K/AKT/MAPK	RVT via regulating the PI3K/AKT/MAPK pathway could inhibit cell proliferation and induce cell apoptosis in chondrosarcoma cells	[95]
Renal cell carcinoma (RCC)	0–100 μM	ACHN, A498, HK-2	N-cadherin, Vimentin, Snail, MMP-2/9, E-cadherin, TIMP-1	AKT, ERK1/2	RVT via inactivating the AKT and ERK1/2 pathways could inhibit proliferation and migration in RCC cells	[96]
Oral cancer	50 μM	CAR, CAL 27	LC3-II/I, Caspase-3/9, Atg-5/7/12/14, Beclin-1, Atg16L1, Apaf-1, AIF, Bcl-2, Bax, Bad	AKT/mTOR, AMPK	RVT via the AMPK and AKT/mTOR pathway could regulate autophagy and apoptosis in cisplatin-resistant human oral cancer CAR cells	[97]
Neuroblastoma (NB)	10–100 μM	SK-N-SH, SH-SY5Y, SK-N-Be2, SMS-KCNR, NB1691	GSK3β, IRS-1, Survivin, PP1α, α-tubulin	AKT	RVT via inactivating AKT by increasing PP1α activity could potentiate 2-DG-induced ER stress and NB cell death	[98]

A clinical study in women with high risk of breast cancer development has shown that serum levels of total trans-resveratrol and glucuronide metabolite are enhanced following consumption of both 5 and 50 mg trans-resveratrol twice daily for 12 weeks. Moreover, this treatment has led to reduction of RASSF-1α methylation parallel with increasing concentrations of serum trans-resveratrol [99].

## Discussion

Several clinical trials have assessed the efficacy, safety, and pharmacokinetics of resveratrol [101]. It has potential beneficial effects in diverse pathological conditions such as diabetes mellitus, obesity, hypertension, neoplastic conditions, Alzheimer's disease, and cardiovascular disorders [101]. However, the therapeutic efficacy of resveratrol seems to be dependent on several factors [102]. For instance, the efficacy of resveratrol has been higher in certain types of cancer compared with others. Moreover, additional clinical trials should be conducted to assess the effects of resveratrol in the treatment of Alzheimer's disease and stroke. Studies in the context of cardiovascular disorders have shown beneficial effects of resveratrol. However, these effects depend on demographics features, since it has not been effective in extremely overweight persons, even has been harmful in schizophrenic patients [103].

Another important note is that the optimal dosage of resveratrol which can induce the maximum beneficial effects without raising toxic effects remains to be identified. A number of studies have reported toxic and adverse effects after consumption of resveratrol [104]. Thus, widespread investigations on the long-term effects of resveratrol in human subjects are needed. Moreover, the interactions between resveratrol and other therapeutic agents should be assessed [104]. A possible adverse effect of resveratrol might be mediated by down-regulation of Akt which induces ROS generation and endothelial cell injury in a dose-dependent manner [105]. Moreover, resveratrol has been shown to alter redox state of human endothelial cells and cause cellular death through a mitochondrial-dependent route [106].

Notably, resveratrol has been found to affect the expression of several genes including cytokine coding genes, caspases, matrix metalloproteinases, adhesion molecules, and growth factors [101]. In addition to the mentioned protein coding genes, evidence from in vitro and in vivo assays has shown the direct effects of resveratrol on several non-coding genes and possible implication of these transcripts in the therapeutic effects of resveratrol [107]. Moreover, it can modulate the activity of several signaling pathways such as PI3K/AKT, Wnt, NF-κB, and Notch pathways [101]. Among the mentioned pathways, the regulatory effects of resveratrol on the activity of the PI3K/AKT pathway have been better appraised in different contexts. In the context of neoplastic conditions, resveratrol not only inhibits malignant behavior of cells and epithelial-mesenchymal transition but also sensitizes neoplastic cells to anti-cancer drugs such as rapamycin [89], doxorubicin [67], vemurafenib [86], cetuximab [66], etoposide [82] and docetaxel [88]. Therefore, it can be used as an adjuvant to enhance the efficacy of several types of anti-cancer modalities ranging from conventional chemotherapeutic agents to targeted therapies. The effects of resveratrol in the suppression of growth of cancer stem cells have been validated in some types of cancers particularly glioblastoma [91]. This property of resveratrol should be appraised in other cancers to find whether it can be used as a drug to combat tumor metastasis and recurrence.

An important issue in the clinical application of resveratrol is the identification of the best route and formulations of this agent. A certain nanoformulation of resveratrol has been proved to be an effective approach for improving the protective effects of resveratrol against lung injury, proposing that the modified-release preparation of this substance can be effective in this situation [49]. Further studies are needed to appraise the efficacy of this formulation in other conditions.

## Conclusion

Taken together, resveratrol has several therapeutic effects including modulation of immune responses and ROS formation, suppression of malignant behavior of cancer cells, and sensitization of these cells to anti-cancer drugs. Increasing the bioavailability of this agent and identification of the most appropriate route of administration of this agent are important changes that should be addressed before the extensive application of resveratrol in clinical settings.

## Data Availability

Data sharing not applicable to this article as no datasets were generated or analyzed during the current study.
